# Hemodynamic characteristics and clinical treatment of patients with iliac vein compression syndrome

**DOI:** 10.3389/fsurg.2025.1542894

**Published:** 2025-07-25

**Authors:** Beihao Shi, Linchen Huang, Sen Wei, Jian Zhu, Zhiwei Li, Jie Ren, Dian Chen

**Affiliations:** Vascular Surgery Department, Affiliated Kunshan Hospital of Jiangsu University, Kunshan, Jiangsu, China

**Keywords:** iliac vein compression syndrome, Cockett syndrome, May-Thurner syndrome, hemodynamics, clinical treatment

## Abstract

Iliac vein compression syndrome (IVCS), also known as May–Thurner syndrome or Cockett syndrome, refers to the compression of the iliac veins by surrounding tissues, typically the compression of the left iliac vein by the right iliac artery anteriorly and the vertebral body posteriorly. A narrowed iliac vein leads to a series of hemodynamic changes that can affect the function of the vascular endothelium and create an environment prone to thrombosis. These hemodynamic parameters can guide stent placement and aid in diagnosing IVCS. This article also summarizes existing methods for the hemodynamic assessment of IVCS and suggests potential future research directions by drawing on the more mature field of arterial hemodynamics.

## 1 Introduction

Iliac vein compression syndrome (IVCS), also known as May‒Thurner syndrome or Cockett syndrome, is a condition in which the iliac veins are compressed by surrounding tissues, most commonly the left iliac vein by the right iliac artery and the vertebral body (1–3). IVCS is more prevalent in women (4), who also have a higher risk of secondary pulmonary embolism. The treatment for thrombotic IVCS is well established and involves thrombolysis followed by balloon dilation and venous stent implantation (5). However, in cases of nonthrombotic IVCS, there is no clear indication for stent placement. This is due to several reasons: (1) Mild compression of the iliac vein is common and does not require treatment, possibly because compression changes the vein from circular to elliptical without affecting the cross-sectional area (6). (2) The severity of stenosis that causes health issues in patients with nonthrombotic IVCS has not been clearly defined, with much of the literature lacking a unified and theoretically grounded definition of “severe stenosis” (2, 7, 8) Therefore, a method to assess the hemodynamics of patients with nonthrombotic IVCS is needed. This is crucial not only to avoid unnecessary endovascular treatments but also to ensure that patients who could benefit are not excluded because of inconspicuous stenosis (9–11). This review comprehensively introduces the methods and indicators for hemodynamic assessment and identifies their potential shortcomings and prospects. Hemodynamic studies on IVCS are summarized (Table 1).

**Table 1:** Study sample and conclusion of included references.

Author	Date	Study sample	Conclusion
Assi et al.	2024	The Subject Group: 4 patients with IVCS and related DVT or lower extremity venous symptoms.	LCIV exhibited elevated shear stress relative to the RCIV and the control group's LCIV. The study suggests that LCIV/RCIV shear stress ratio could serve as an effective metric for risk stratification in IVCS patients.
		The Control Group: 4 patients with arterial disease, but without venous diseases.	
Assi et al.	2023	Subject1: 1 patient with IVCS and related DVT.	The study presents a standardized protocol to assess hemodynamics in IVCS.
		Control1: 1 patient with carotid artery disease.	Shear rates increase at the stenosis of the left iliac vein.
Changsheng et al.	2023	1 patient who underwent percutaneous angioplasty	Wall shear stress (WSS) is elevated at the stenosis site and outside the intersection area.
		14 models with different morphologies were established based on the real model of the patient.	An increase in the taper angle and a smaller tilt angle can lead to adverse hemodynamic events.
Chen et al.	2022	69 chronic venous disease (CVD) patients, with a total of 128 limbs studied.	Reduced iliac venous flow and stenosis severity >50% may indicate better outcomes of endovascular treatment.
Chen et al.	2023	31 patient-specific iliac venous CFD models	The peak velocity ratio across the compression site and the angle between the left and right iliac veins can be used together to determine whether IVCS is present.
Delis et al.	2007	16 patients (23 limbs) with advanced Chronic	Stent implantation can relieve the symptoms of venous claudication and boost the calf muscle pump function.
		Venous Disease due to chronic iliofemoral and inferior vena cava thrombosis	Stent implantation also leads to an increase in the degree of venous reflux
Fan et al.	2024	The CFD study is based on a patient-specific model	Stent implantation depth can cause adverse hemodynamic events;Implanting high-density strut stents may lead to adverse hemodynamic events.
		In the *in vitro* experiment, a transparent glass model is utilized to mimic the geometry of the iliac vein.	By discussing Pascal's law, Torricelli's law, Bernoulli's law, and Poiseuille's law, the article explains how these fluid laws link to the anatomy and physiology of veins.
Guven	2024	N/A	
Hu et al.	2024	The study uses an idealized iliac vein model, based on general anatomical data	Stent implantation depth can cause adverse hemodynamic events; The crown—shaped Vena Stent has a better hemodynamic advantage than other stents.
Jiang et al.	2021	15 patient-specific IVCS models	The predicted blood stasis shows a higher correlation with The predicted blood stasis shows a higher correlation with clinically observed DVT.
Jiang et al.	2023	Four stenosis models based on an idealized model.	Iliac vein stenosis of 70% is more likely to be clinically significant compared to other degrees of stenosis.
Jian et al.	2024	A 61-year-old female patient with IVCS	Bilateral and left hip flexions increase the risk of thrombosis.
			Active ankle exercise and intermittent pump compression therapy can effectively improve these conditions.
Jun	2019	35 patients with DVT associated with IVCS	The presence of mild iliac vein stenosis in IVCS can predict
			TBIVS and indicates a need for closer monitoring for PE.
Kurstjens et al.	2017	The study included 196 patients with 237 limbs, of whom 39 patients with 45 limbs underwent stenting.	Venous occlusion air plethysmography cannot identify venous obstruction proximal to the femoral confluence or distinguish patients who will benefit from stenting.
Kurstjens et al.	2014	4 patients with post-thrombotic deep venous obstruction of the iliofemoral tract.	Pressure measurements of the common femoral vein, might be able to identify a significant outflow obstruction due to post-thrombotic disease.
Kurstjens et al.	2016	N/A	Plethysmography seems not to be valuable in predicting the outcome of endovascular therapy.
			Reduced inflow into the common femoral vein seems to predict in-stent stenosis or occlusion.
Kurstjens et al.	2018	12 patients with unilateral post-thrombotic iliofemoral obstruction.	Stenting of post-thrombotic iliofemoral obstruction reduces venous hypertension and improves quality of life.
Kurstjens et al.	2015	14 patients with 14 diseased limbs and 14 control limbs	The development of collateralisation can limit the pressure, but it doesn't eliminate the need for treatment.
Lattimer and Mendoza	2016	33 patients were divided into three groups with each group consisting of 11 subjects (11 legs).	VDI was significantly reduced in patients with iliac and femoral obstruction.
Li et al.	2022	Iliac vein model established based on the normal human iliac vein.	The corolla design of the front end of the stent helps reduce the disturbance to contralateral hemodynamics
			Implantation at the left branch outlet has the least influence on the blood flow of the iliac vein
Li et al.	2023	A patient with IVCS who underwent preoperative and postoperative CTV tests.	Stent placement can relieve clinical symptoms in short term, but may increase the risk of thrombosis in long term.
Neglén et al.	2003	447 limbs underwent iliac vein stenting.	Clinical improvement can be achieved after iliac vein stenting, regardless of whether venous reflux is present.
Oğuzkurt et al.	2007	A female patient presented with recent left lower extremity swelling.	Duplex scanning observed a sharp increase in blood flow velocity at the compression site, rising from 40 cm/min to 100 cm/s.
Pei et al.	2024	24 subjects with nonthrombotic iliac vein lesions (NIVLs) were included.	Some objective and quantitative indicators (Δ*P*, Δ*V*, etc.) have been summarized to assess the severity of NIVLs patients’ conditions.
Wang et al.	2022	11 patients with IVCS	The pressure differenc becomes larger with an increase in the degree of stenosis and the confluence angle.
			Low TAWSSand high RRT area near the compression site may increase the risk of thrombosis.

## 2 Altered hemodynamics in iliac vein compression syndrome

**Figure 1:**
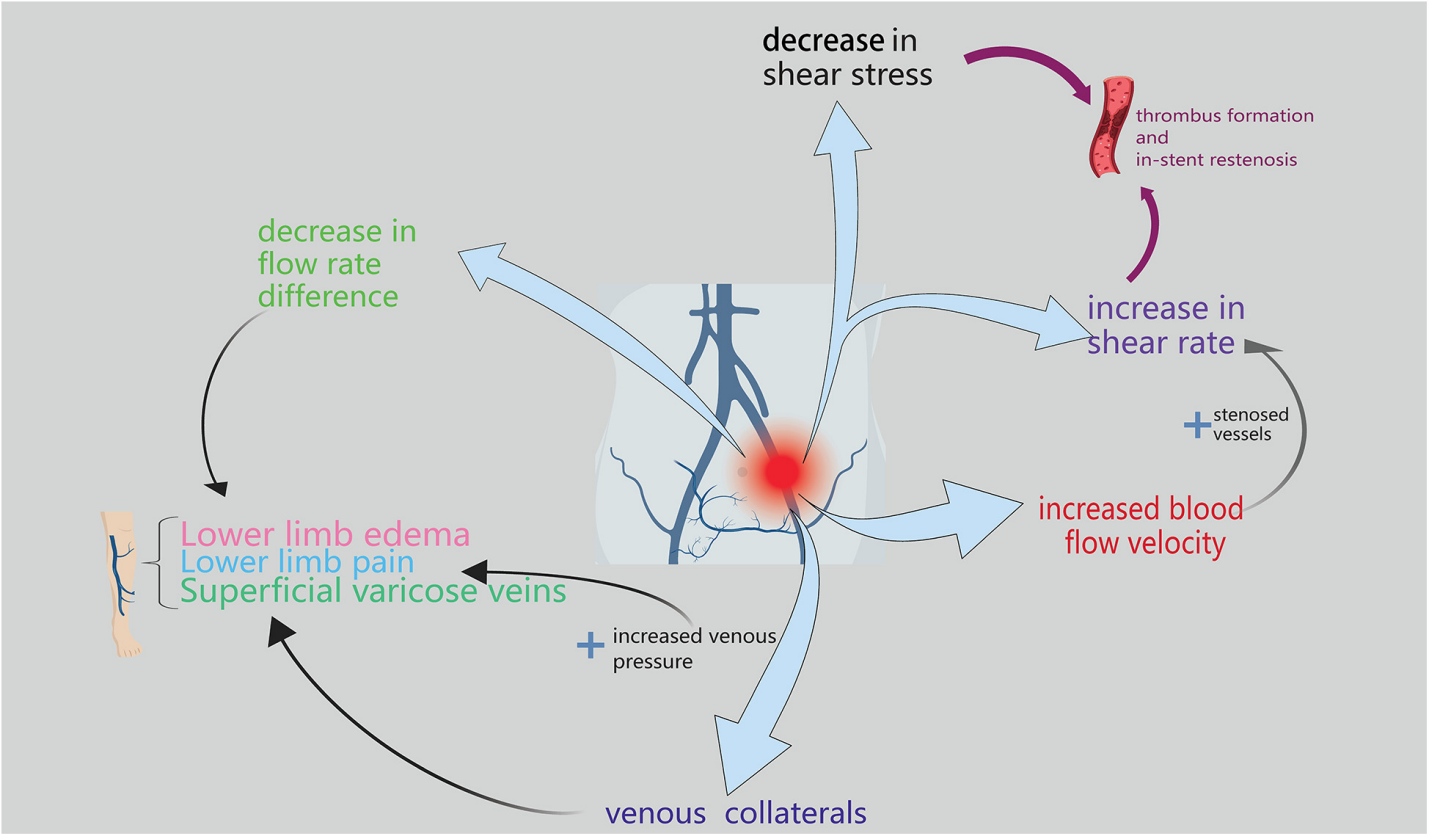
The figure primarily depicts the hemodynamic manifestations of lliac vein compression syndrome, which are mainly reflected in flow rate difference, venous pressure, blood flow velocity, shear rate, and shear stress.

The changes in hemodynamics and common parameters used to assess such changes have been described (Figure 1). These parameters can be broadly divided into two categories: parameters that can obtained via traditional imaging examinations and parameters that require additional tools for measurement or simulation.

### 2.1 Hemodynamic parameters obtained from traditional imaging examinations

Currently, the diagnosis of iliac vein compression syndrome (IVCS) relies mainly on imaging methods such as venography, CT venography (CTV), and intravascular ultrasound (IVUS). Each of these methods has its own advantages. Venography, a traditional vascular examination method, is considered the gold standard for the diagnosis of IVCS. Its strength lies in the ability to clearly observe the stenosis of the iliac vein and the presence of collateral circulation, as well as to identify whether there is thrombus formation. CTV, which involves the injection of contrast agents, can obtain clear images of the affected blood vessels. Its advantage is the provision of clearer and intuitive images. Moreover, through vascular modeling and hemodynamic simulation, more detailed and abundant hemodynamic parameters can be obtained. IVUS is a technique in which an ultrasound probe is placed inside a blood vessel. It plays an increasingly important role in the diagnosis and treatment of IVCS. IVUS not only has high sensitivity and specificity for the diagnosis of IVCS but can also accurately measure the diameter of blood vessels to guide the selection of iliac vein stents. The acquisition of all hemodynamic parameters is inseparable from the support of the abovementioned imaging examinations (12).

#### 2.1.1 Blood flow velocity (BFV)

Changes in blood flow velocity can be easily observed with ultrasound. Ultrasound studies have shown that the blood flow velocity at the site of stenosis increases by at least 3–8 times according to Bernoulli's law (Bernoulli's law) (13–17). Studies have shown that both the *Δ*V (the difference in blood flow velocity between the stenotic segment of the left common iliac vein and the confluence of the left internal and external iliac veins) and the blood flow velocity in the stenotic LCIV segment are positively correlated with the clinical classification (C grade) in the CEAP classification system and may serve as indicators for evaluating iliac vein stenosis (18). In a simplified model, the shear rate can be estimated with the following formula: shear rate (SR) = (*V* × *D*)/2, where *V* is the velocity and *D* is the vessel diameter. It can be inferred that as the blood flow velocity in patients with IVCS increases, the shear rate at the site of stenosis also increases (19) because the increase in flow velocity is often greater than the reduction in vein diameter.

#### 2.1.2 Flow rate (FR)

The flow rate can be inferred from the BFV (19). Simply assessing the flow rate at the site of stenosis has limited significance, and most researchers further process and analyze the flow rate. Chen et al. studied the difference in FR between the common iliac vein (CIV) and the external iliac vein (EIV). A difference in FR less than 0 ml/s indicates that the stenosis has significantly reduced the flow rate of the proximal CIV. The difference in FR is negatively correlated with the patient's clinical symptoms (Pearson's correlation, *r* = −0.44, *P* < 0.001) and can further predict the benefits of endovascular treatment, which will be described in detail later (20). Lattimer CR and colleagues used air plethysmography (APG) to investigate the significance of blood flow. This technique indirectly reflects blood flow by measuring changes in the volume of the lower leg and essentially represents the efficiency of venous return. Two main parameters were measured: (1) Venous Drainage Index (VDI): the rate at which the volume of the lower leg decreases from a dependent position (with the leg lowered) to an elevated position (with the leg raised). (2) Venous Filling Index (VFI): the rate at which the volume of the lower leg expands when it is raised to a dependent position. This index reflects the speed at which blood flows into the lower limb venous system under the influence of gravity. The results showed that these two hemodynamic parameters can be used to assess venous obstruction and reflux effectively: the critical value for VDI to distinguish obstruction was 10.8 ml/s (*P* < 0.0005), and the critical value for VFI to distinguish reflux was 2.9 ml/s (*P* < 0.0005). This is a noninvasive method for quantifying reflux and obstruction in patients with iliac vein compression syndrome in clinical settings. This method may also help to assess the impact of venous stenting on hemodynamics (21). However, a study by Kurstjens and colleagues revealed that APG cannot ascertain which patients will benefit from stent placement (22), as described in the following text.

#### 2.1.3 Collateral vessels

Assessing the condition of collateral vessels is also an important part of the hemodynamics of patients with IVCS. The collateral circulation mainly reflects the following: (1) Severity of obstruction: patients with abundant iliac vein collaterals usually have more significant stenosis. (2) Location of the obstruction: the position and direction of the collaterals can infer the location of the stenosis. (3) Abundant collateral circulation can alleviate severe symptoms after thrombosis. Thomas et al. divided the collaterals of the iliac vein into three groups: collaterals for external iliac vein obstruction, collaterals for common iliac vein obstruction, and collaterals for combined obstruction of the external and common iliac veins. Through this grouping, surgeons can infer the location of the stenosis (23). The presence of abundant collaterals also implies that the patient's clinical symptoms can be partially relieved. In a study involving balloon occlusion of a normal external iliac vein, the median femoral vein pressure increased from 12.8 mmHg to 23.5 mmHg. In comparison, the median femoral vein pressure in patients with IVCS was 17.0 mmHg. This finding indicates that establishing collateral circulation can partially compensate for the narrowed and obstructed iliac vein (24). Complete compensation would require an exponential number of collaterals; for instance, a completely obstructed 16 mm common iliac vein must be compensated by 256 collaterals, each being 4 mm in size (20). This finding indicates that quantifying the degree of compensation using imaging and then using the degree of compensation to assist in determining endovascular treatment is challenging. Therefore, the richness of venous collaterals can be used for the diagnosis of IVCS (25), but accurately assessing the benefits of endovascular treatment is not possible.

#### 2.1.4 Iliac vein morphology

Morphological research on iliac vein compression syndrome (IVCS) focuses on two key areas: the morphology of the stenosis and that of the iliac vein itself. With respect to stenosis morphology, studies have revealed a significant positive correlation between stenosis length and both thrombus formation outside the narrowed iliac vein (TBIVS) and the clinical classification (C grade) in the CEAP classification system (18, 26). Moreover, when the compressed iliac vein is near the confluence point, hemodynamic parameters such as shear stress, pressure difference, and velocity change abruptly, increasing the likelihood of thrombosis. Recent research has also revealed a negative correlation between the minimum cross-sectional area of the stenosed left iliac vein segment and the cross-sectional area distal to the stenosis and the clinical classification (18). With respect to iliac vein morphology, the confluence angle between the left and right iliac veins is moderately positively correlated with the pressure difference between the start and end of stenosis [Pearson's correlation coefficient (*r*) = 0.638] (27). Additionally, a smaller inclination angle of the left iliac vein and a larger iliac vein taper angle can lead to adverse hemodynamic events (18, 28). Research has more deeply investigated the inclination angle of the left iliac vein and revealed that blood flow disturbances significantly increase when the angle angle is less than 120 degrees (29).

### 2.2 Hemodynamic parameters obtained via computational fluid dynamics and particle image velocimetry

Computational fluid dynamics (CFD) and particle image velocimetry (PIV) can obtain hemodynamic parameters that are not accessible through conventional imaging methods after models of the vasculature and blood are established (30).

Computational fluid dynamics (CFD) is increasingly being employed to obtain hemodynamic parameters. The CFD studies cited in this paper are summarized (Table 2). Typically, researchers use commercial or open-source software (e.g., Mimics, SimVascular) to extract imaging data and create models. Common imaging methods include computed tomography (CTV) and magnetic resonance (MR), with thin-slice and high-quality images being optimal for model accuracy. After an iliac vein model is generated, Ansys meshing is commonly used for mesh generation, followed by hemodynamic simulation with Ansys Fluent. Some medical parameters, such as the time-averaged wall shear stress (TAWSS) and oscillatory shear index (OSI), require specialized software (e.g., CFD-post, Tecplot) for analysis (31, 32).

**Table 2:** Imaging data and boundary conditions of included CFD studies.

Author	Date	Imaging data	Boundary conditions
Assi et al.	2024	CT, MR, US	Inlet: Extracted from Ultrasound, Respiratory Cycle Setting
			Outflow: A 3-element Windkessel lumped-parameter model
			Wall Boundary Conditions: Set as rigid wall without slip.
Assi et al.	2023	CT, US	Inlet: Extracted from Ultrasound, Respiratory Cycle Setting
			Outflow: A 3-element Windkessel lumped-parameter model
			Wall Boundary Conditions: Set as rigid wall without slip.
Changsheng et al.	2023	CTA	Inlet: The iliac vein velocity curve was used as the inlet velocity.
			Wall Boundary Conditions: Set as rigid wall without slip.
Chen et al.	2023	CT	Inlet: The inlet velocities on the left and right sides were assigned as 0.03 m/s
			and 0.12 m/s, respectively.
			Outlet: The blood flow velocity in the inferior vena cava was set at 0.06 m/s.
			Wall Boundary Conditions: Set as rigid wall without slip.
Fan et al.	2024	CT	Inlet: A pulsatile velocity waveform obtained from a healthy individual at rest
			Outlet: An unstable pressure
			Wall Boundary Conditions: Set as rigid wall without slip.
Hu et al.	2024	N/A	Inlet: an instantaneous blood flow velocity from references
			Outlet: Zero-pressure boundary conditions
			Wall Boundary Conditions: Set as rigid wall without slip.
Jiang et al.	2021	CTV	Inlet: Extracted from Ultrasound
			Outlet: Zero-pressure boundary conditions
			Wall Boundary Conditions: Set as rigid wall without slip.
Jiang et al.	2023	N/A	Inlet: Acquired from previous literature
			Outlet: Zero-pressure boundary conditions
			Wall Boundary Conditions: Set as rigid wall without slip.
Jian et al.	2024	CTA	Inlet: Changed according to different movement patterns.
			Outlet: The outlet condition was adopted at the IVC.
			Wall Boundary Conditions: Set as rigid wall without slip.
Li et al.	2022	N/A	Inlet:iliac vein velocity curve
			Outlet: Set as pressure outlet.
			Wall Boundary Conditions: Set as rigid wall without slip.
Li et al.	2023	CTV	Inlet: derived from ultrasound and assumed to be constant.
			Wall Boundary Conditions: Set as rigid wall without slip.
Pei et al.	2024	CT	Inlet: Extracted from Ultrasound
			Outlet: the outlet was set 1 cm below the lower margin of the left renal vein
			Wall Boundary Conditions: Set as rigid wall without slip.
Wang et al.	2022	CTV	Inlet: Extracted from clinical ultrasound images
			Outlet: Set at the venous blood downstream (outlet) of IVC
			Wall Boundary Conditions: Set as rigid wall without slip.

Particle image velocimetry (PIV) is an experimental fluid dynamics technique. This involves constructing a 3D-printed iliac vein model, seeding the fluid with tracer particles, illuminating the flow field with a laser sheet, and capturing particle motion with a high-speed camera to calculate velocity distributions and related parameters. PIV is highly important in iliac vein compression syndrome research. Its key advantage lies in acquiring hemodynamic parameters within a real iliac vein model, offering more intuitive and detailed data. However, PIV is costly and challenging to apply clinically, making it more suitable for validating CFD results in scientific research (31, 33).

#### 2.2.1 Shear rate

The shear rate is closely related to the formation of secondary thrombi. The shear rate refers to the rate of change in velocity in the direction perpendicular to the blood flow velocity. A study revealed that during the process of arterial thrombus formation, an increased shear rate (>1,000 s^−1^) causes red blood cells to shift toward the central blood flow layer, which in turn increases the concentration of platelets near the vessel wall (34). Von Willebrand factor (VWF) initiates platelet aggregation under conditions of high shear stress independent of activation (35). Finally, these cells adhere to the vessel wall after narrowing, forming a thrombus. Therefore, studying the shear rate is crucial for understanding thrombosis in patients with IVCS. Studies have shown that patients with IVCS have higher shear rates in the stenotic area (9, 36). Moreover, the shear rate is constantly changing. The shear rate is not only affected by factors such as hydration status, muscle condition, cardiac function, and anatomical issues on a given day but also influenced by the current thrombotic state of the limb [thrombus recanalization, thrombus extension, recurrent thrombosis, and new thrombus formation (37)]. Cyclic variations are observed: an increase in the early shear rate leads to thrombus formation, followed by an increase in resistance to thrombus formation and a decrease in shear rate, eventually resulting in thrombus recanalization and a subsequent increase in shear rate as the cycle repeats (9). Even within the same limb, the shear rate of the common iliac vein (CIV) varies at different time points. To minimize the interference of these factors, Assi and colleagues introduced the left common iliac vein (LCIV)-to-right common iliac vein (RCIV) shear rate ratio, which is particularly applicable for patients with unilateral iliac vein compression. The mean LCIV/RCIV shear rate ratio was 1.43 ± 0.6 in the control group and 6.56 ± 0.9 in the subject group (*p* = 0.00008), making it a standardized and interpretable assessment indicator (9).

#### 2.2.2 Shear stress

Shear stress is the tangential force exerted on the blood vessel wall during blood flow. This indicator has a more pronounced effect on the blood vessel wall than the shear rate does and has a significant effect on thrombus formation and vascular endothelial function. Studies have shown that the TAWSS is high at the site of stenosis (with a maximum value of 2.0 Pa, while the normal range is 0.6–1.0 Pa) and lower upstream and downstream of the site of stenosis (27). Furthermore, research employing an idealized iliac vein model has examined the relationship between stenosis and wall shear stress (WSS). The study revealed that in the 70% stenosis model, the change in the WSS was more significant, with the WSS value in the stenotic region reaching 4.60 Pa, which is twice that of the 50% stenosis model (38). Recently, studies have shown that the TAWSS is positively correlated with the clinical CVI classification and may help evaluate the clinical progression of lower limb venous insufficiency in IVCS patients (18).

At present, the shear rate and shear stress in patients with IVCS can be simulated only through CFD and PIV, which limits their application in certain scenarios and makes them difficult to use clinically. Owing to the increase in computing power, research on the shear rate and shear stress in patients with iliac vein compression syndrome will likely become more frequent, and their relationship with disease progression will be studied more thoroughly.

## 3 Endovascular therapy and hemodynamic parameters

**Figure 2:**
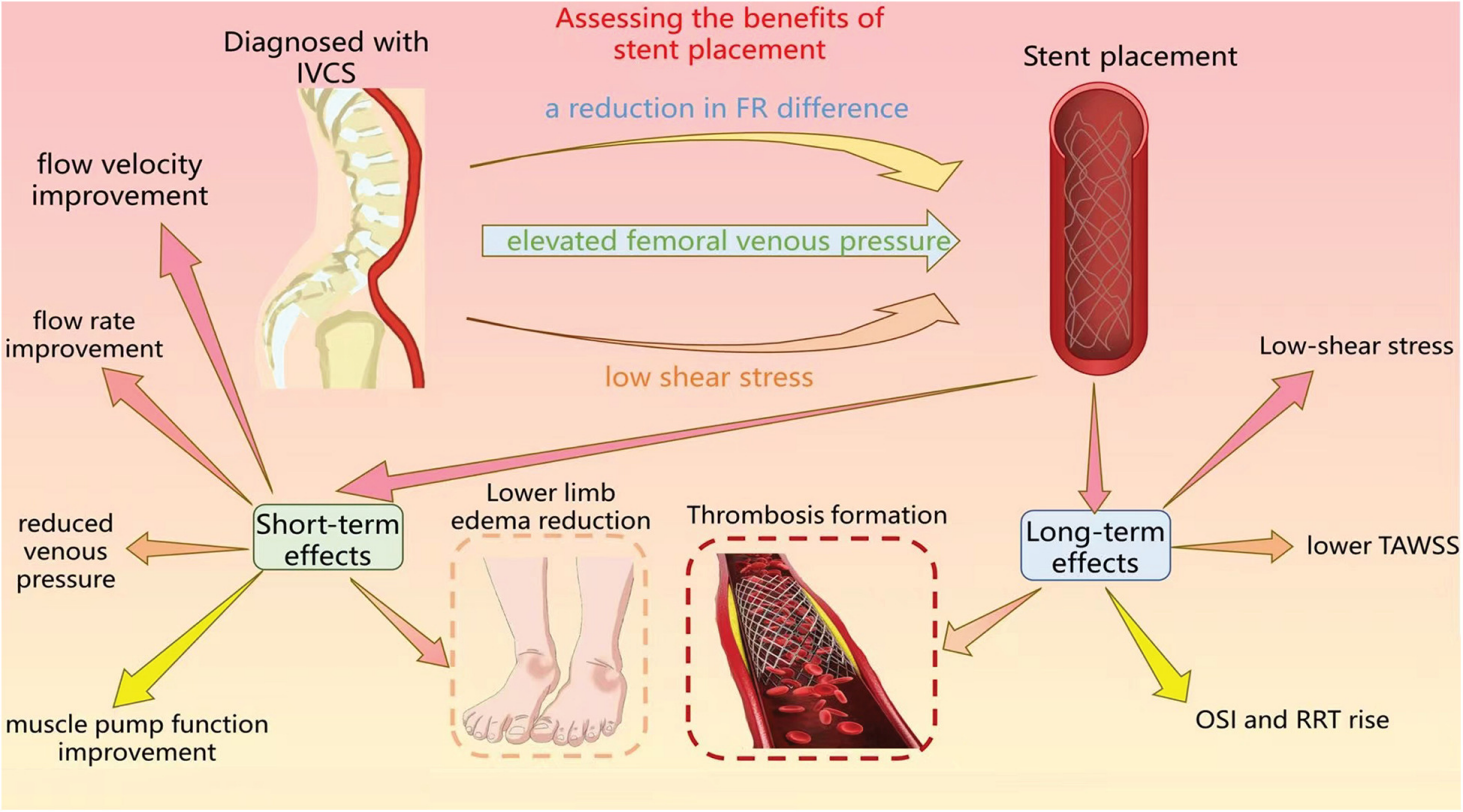
The figure primarily delineates the utilization of hemodynamic assessment to evaluate the benefitd of stent implantation, as well as the short-term and long-term impacts subsequent to the procedure.

As shown in Figure 2, the relationship between changes in hemodynamic parameters and vascular treatment have been explained.

### 3.1 Hemodynamic parameters related to endovascular therapy

#### 3.1.1 Fr difference

In patients with IVCS, if the difference in FR is negative (<0), the stenosis affects the flow of the iliac vein. In this case, endovascular treatment is likely to effectively relieve symptoms. Conversely, if the difference in FR is positive (≥0), the stenosis at the patient's iliac vein may not affect their overall health, and the benefits of endovascular treatment in this situation are relatively limited (20).

#### 3.1.2 Air plethysmography (APG)

The parameters measured via APG help assess the effectiveness of venous stent placement (21). In a previous study, researchers used the APG to assess three hemodynamic parameters: one-second outflow volume, total venous volume, and outflow fraction. The AUC values of these three indices are low, whether for identifying post-thrombotic obstruction (0.71/0.69/0.59, respectively) or for determining the success of stent implantation (0.57/0.54/0.63, respectively), and these indices cannot be used clinically (22). In a systematic review, researchers reached the same conclusion as mentioned above, and further development of new hemodynamic assessment methods is needed (39).

#### 3.1.3 Venous pressure

According to Pascal's Law, any change in pressure at one point in the venous system is transmitted equally throughout the entire system, so the venous pressure in the lower limbs can reflect the pressure conditions of the iliac veins (16). Femoral venous pressure may help determine whether patients with thrombotic iliac vein compression syndrome require endovascular treatment, as it can indirectly reflect the degree of obstruction caused by post-thrombotic syndrome. However, because these studies were small-sample studies, further research is needed to clarify the relationship between venous pressure and hemodynamics (39–41). Dorsal foot vein pressure measurements were taken, and the study revealed no significant difference between the control limb and the affected limb. However, these studies were small-sample studies, so further research is needed to clarify the relationship between venous pressure and hemodynamics (40). Recently, studies have shown that both the pressure difference between the two ends of a stenotic segment (*Δ*P) and the pressure at the caudal end of the stenotic LCIV segment are positively correlated with the clinical classification (C grade) in the CEAP classification system (18) and may serve as indicators for assessing iliac vein stenosis.

#### 3.1.4 Shear stress

Shear stress is uniquely advantageous for describing recurrent stenosis and thrombosis after stent implantation. An animal experiment involving the implantation of a stent in the iliac artery has indicated that increased shear stress in blood vessels is beneficial in that it reduces inflammatory responses, decreases the migration of smooth muscle cells, and prevents rupture of the elastic membrane (42). In contrast, a study on coronary stent thrombosis and restenosis has shown that low endothelial shear stress (ESS) is strongly associated with in-stent restenosis and thrombosis (43). If there is a wide area of low shear stress at the junction and opening of the iliac vein bifurcation, the risks and benefits of endovascular stenting should be carefully assessed (44, 45). However, there are some differences between venous and arterial diseases. A study has indicated that in patients with iliac vein compression syndrome, there is no significant correlation between the WSS values at the stenosis site and the risk of deep venous thrombosis (DVT). Thus, WSS cannot be used alone as a reliable indicator for assessing the risk of DVT (38).

#### 3.1.5 Old blood volume fraction

Old Blood Volume Fraction (OBVF) is a metric for evaluating the degree of blood stasis. One study revealed a high consistency between blood stasis assessed by OBVF in patients with iliac vein compression syndrome and clinically observed DVT, aiding doctors in preoperatively assessing DVT risk and guiding endovascular therapy more accurately (46). Another study established an idealized iliac vein model and demonstrated that 70% iliac vein stenosis resulted in significant blood stasis, which is closely associated with DVT development (38).

### 3.2 Hemodynamic changes after stent implantation

Stent placement for hemodynamic stabilization can lead to short-term symptom relief as well as long-term prevention of recurrent stenosis or thrombosis (47).

#### 3.2.1 Short-term effects

In terms of flow velocity, the regurgitation time and peak regurgitation velocity decreases (*P* < 0.01), whereas the average flow velocity increases (*P* < 0.05) (48). In terms of flow rate and venous pressure, after stent implantation, the first-second flow fraction and residual volume fraction significantly improved, the total femoral venous pressure significantly decreased, and muscle pump function in the lower leg improved, as evidenced by increases in the ejection volume (EV) and ejection fraction (EF) (49). Although some studies revealed that venous reflux may worsen after stent placement [as evidenced by a venous filling index (VFI) value greater than that of the control group] (49), other studies suggest that it does not worsen (50). These hemodynamic changes lead to improvements in clinical symptoms, as evidenced by the regression of lower limb edema.

In the PIV experiment, stent implantation led to a rightward deviation in the direction of blood flow, and local low-speed blood flow was observed, but it did not significantly affect the overall flow field (51, 52), indicating that early after stent placement, there were no significant harmful hemodynamic changes.

#### 3.2.2 Long-term effects

These studies have focused mainly on in-stent restenosis and thrombosis, both of which are caused by long-term low-shear stress and changes in flow velocity after stent implantation. Studies evaluating arterial bifurcations have shown that stent placement not only leads to a decrease in blood flow velocity but also increases the area of low shear stress regions (53, 54). Studies on IVCS have shown that deeper stent placement and denser stent struts can have a greater impact on the flow field, leading to the generation of vortices (especially when the front end of the stent is implanted more than 20 mm deep into the iliac vein outlet). Deeper stents can also significantly reduce the time-averaged wall shear stress (TAWSS) of the iliac vein (31, 55) and increase the oscillatory shear index (OSI) and relative residual time (RRT) (33). Thick and malpositioned stents can cause an already poor hemodynamic status to worsen (32, 55). Similar scenarios can also occur in the placement of arterial stents (45, 56).

Additionally, in a study, researchers conducted in-depth analyses of different motion models for patients with stents. The results revealed that left hip flexion and right hip flexion significantly disturb the flow field, the supine position, despite showing irregular flow, is relatively better than a position in which the hip is flexed, and blood flow within the stented segment is continuous in the sitting position. Furthermore, the relationships among wall shear stress (WSS), OSI, and different motion states have been clarified: (1) WSS: the sitting position and left hip flexion can exacerbate low shear stress at the stent support site; (2) OSI: the OSI value is high mainly at the junction of the stent tail end and the iliac cavity, with the highest OSI values in the sitting and supine position models (32).

Studies have shown that, owing to its design, a corolla iliac vein stent has a relatively small effect on hemodynamics. Because of its design, this stent can avoid adverse hemodynamic changes caused by stent placement, such as low shear stress areas and flow disturbances, which are associated with increased risks of restenosis and thrombosis (31, 55). Additionally, research has shown that an increase in the number of stent struts leads to the deterioration of hemodynamic parameters (57). This finding indicates that an optimized stent design will have minimal impact on hemodynamics. These findings indicate that an optimized stent design has a minimal effect on hemodynamics.

## 4 Conclusion

This article mainly summarizes the hemodynamic changes associated with IVCS, how to judge whether endovascular treatment is needed as a result of these changes, and new hemodynamic changes that may occur after endovascular treatment. The main purpose of this study is to provide new auxiliary references for clinical surgeons to decide whether to perform endovascular treatment.

We have summarized the imaging methods used in existing CFD studies (Table 2) and highlighted the advantages and disadvantages of each. Currently, CFD studies rely primarily on imaging data from CTV; however, the potential benefits of IVUS for CFD research should not be overlooked.

The existing CFD studies on iliac vein compression syndrome have several limitations: (1) Simplified vessel geometry: Most studies assume rigid iliac vein walls, which may introduce deviations in the CFD results. (2) Idealized physiological conditions: Most studies overlook the impact of physiological activities (e.g., breathing, cardiac cycles, and body position) on hemodynamics, despite their significant influence on the venous system (36, 39). (3) Inaccurate boundary conditions: Boundary conditions (e.g., flow, velocity waveforms, pressure) are often not measured experimentally but are instead referenced from prior studies. (4) Importantly, no studies have compared the diagnostic efficacy between CFD and phlebography. Moreover, most studies included patients in CFD research did so only after the patients were diagnosed with IVCS through phlebography. Therefore, whether CFD can be used as a standalone IVCS diagnostic method remains uncertain. Furthermore, notably, no studies have applied IVUS in CFD research, which represents a significant limitation. In fact, IVUS may offer several advantages for CFD studies. First, the real-time imaging capabilities of IVUS allow more accurate vascular modeling. The static images from CTV can often make it difficult for researchers to distinguish whether the contrast agent deficiency is due to iliac vein stenosis or the impact of blood flow from the contralateral iliac vein. With IVUS, however, the internal structure of the patient's veins can be clearly and dynamically monitored. Second, IVUS can provide more realistic boundary conditions. Most current CFD studies defined the vessel wall as rigid, ignoring its thickness, elasticity, and some fine structures (such as webs, trabeculations, and spur morphologies) and affecting the accuracy of CFD studies. IVUS, in contrast, can be used to accurately measure the thickness of the vessel wall, assist in determining elasticity, and identify potential fine lesions, thereby further optimizing the structures within the boundary conditions (58). Finally, IVUS can be used to accurately determine the degree of vascular stenosis and guide selection of the diameter, length, and position of the stent (12). No existing CFD studies related to stent placement incorporated IVUS as an effective assessment tool.

In future research, the use of computational fluid dynamics and particle image velocimetry (PIV) technology to understand the hemodynamic changes in patients with IVCS is very promising, however, limited research has been conducted in this regard. More large-sample studies are needed to establish a strong correlation between parameters such as shear rate and shear stress and the progression of IVCS.
